# Psychometric Characteristics of Oral Pathology Test Items in the Dental Hygiene Curriculum—A Longitudinal Analysis

**DOI:** 10.3390/dj9050056

**Published:** 2021-05-13

**Authors:** Mythily Srinivasan

**Affiliations:** Department of Oral Pathology, Medicine and Radiology, Indiana University School of Dentistry, Indiana University Purdue University at Indianapolis, Indianapolis, IN 46202, USA; mysriniv@iu.edu

**Keywords:** dental hygiene, oral pathology, exam soft, item analysis

## Abstract

As the landscape of oral healthcare and the delivery of services continue to undergo change, the dental hygienist plays an increasing role in assisting dentists with oral diagnosis and preventive strategies. Hence, the dental hygiene curriculum standards require biomedical science instructions, including general and oral pathology. Student learning and cognitive competencies are often measured using multiple-choice questions (MCQs). The objectives of this study were to perform a longitudinal analysis of test items and to evaluate their relation to the absolute grades of the oral pathology course in the dental hygiene curriculum. A total of 1033 MCQs covering different concepts of oral pathology administered from 2015 through 2019 were analyzed for difficulty and discriminatory indices, and the differences between the years were determined by one-way ANOVA. Test reliability as determined by the average KR-20 value was 0.7 or higher for each exam. The mean difficulty index for all exams was 0.73 +/− 0.05, and that of the discriminatory index was 0.33 +/− 0.05. Wide variations were observed in the discriminatory indices of test items with approximately the same difficulty index, as well as in the grade distribution in each cohort. Furthermore, longitudinal data analyses identified low achieving cohorts amongst the groups evaluated for the same knowledge domain, taught with the same instruction, and using similar test tools. This suggest that comparative analyses of tests could offer feedback not only on student learning attributes, but also potentially on the admission processes to the dental hygiene program.

## 1. Introduction

Dental hygienists play an integral role in assisting individuals and groups in achieving and maintaining optimal oral health. Thus, the dental hygiene educational guidelines recommended by the Commission on Dental Accreditation (CODA) require instructions on biomedical sciences to ensure an understanding of the basic biological principles for comprehensive oral hygiene care [1]. The CODA standards specify that pathology class time hours should be classified in terms of general pathology and oral pathology. By description, the general pathology content areas focus on the nature of disease processes and the associated alterations in structure and function.

The oral pathology content emphasizes the etiopathogenesis of oral diseases, and the systemic pathology teaches the etiologies and host responses of organ systems [2]. Traditionally, dental hygiene education has relied on a teacher-delivered, lecture-based curriculum and a performance-based approach to clinical activities. In recent years, the lecture as an instructional format is supplemented with a variety of useful adjunct educational tools, such as videos, student-led discussions, and online activities that are incorporated into the curriculum. This ensures the proper transfer and acquisition of knowledge, preparing the students to understand and participate comprehensively in the delivery of oral healthcare [3].

Student learning is often evaluated using multiple choice questions (MCQs) that test cognitive competencies [4]. The assessment of learning is an important element of an instructional design process, which provides feedback on learning and teaching processes and enables the review and improvement of the whole process [5,6]. There have been few reports on the assessment of general and oral pathology instruction in terms of instructional content and student performance [7]. Various methods are used to assess multiple-choice tests to provide feedback on learning and teaching processes. Item analysis is one such method that examines student responses to individual test item. The Difficulty Index (DI) is the percentage of students who chose the correct answer, and is expressed as a fraction of 1 or as a percentage. The Discrimination Index (Disc-I), or point biserial correlation, measures how students who did well or poorly overall performed on an item. In other words, the discriminating measures evaluate how performance on a single item correlated with overall performance [8,9,10].

This study aims to determine the DI, Disc-I, or point biserial correlation of the MCQs administered as part of the oral pathology course in the dental hygiene bachelor’s degree program offered through Purdue University at Indianapolis, Indiana. The MCQs were designed to test the student’s comprehension of the content and its application to the practice of dental hygiene. The specific research objectives were to perform item analysis of MCQ test items in an oral pathology course to evaluate the relationship between the DI and Disc-I of multiple-choice questions and the distribution of grades in the oral pathology course in the dental hygiene curriculum; and (2) to compare the reliability of the MCQ exams assessing the same knowledge domain across multiple years.

## 2. Methods

Question cohort and participants: The study cohort consisted of 1033 MCQs (with four choices) that were included across twenty exams in the fall semesters of 2015–2019 at four exams per year, covering different concepts of oral pathology. The number of exam takers were 30 in each exam in 2015, 27 in each exam in 2016, 19 in each exam in 2017, and 20 in each exam in 2018 and in 2019.

Data collection: ExamSoft testing software (ExamSoft Worldwide, Dallas, TX, USA) was used to administer the MCQ exams [11]. Questions were presented as one question per screen. The exam takers were allowed one hour to complete the exam, and could advance to the next question, review previous questions, and change answers as desired. After completion, the exam takers uploaded the examination file to the ExamSoft database. All questions in each exam were used for data collection, and the raw score of each exam taker in terms of the total number of correct responses, the percentage of correct responses, and the letter grade based on a pre-determined range were obtained in the summary report. In ExamSoft, the internal consistency and reliability of each exam was measured by KR-20 (Kuder–Richardson Formula). It considers all dichotomous questions and how many exam takers answered each question correctly [12]. The ExamSoft statistical report for each item also included DI, Disc-I, and point biserial.

Data analysis: The mean DI, Disc-I, and point biserial were calculated for each of the four exams of each year. The difference in the mean scores of DI, Disc-I, and point biserial scores was assessed by one-way ANOVA and Tukey’s post hoc analysis. A *p*-value of less than 0.05 was considered significant. An absolute grading system was used to provide a letter grade for the exam based on the average scores of all four exams at the end of the semester in each year on a scale of 90–100 points for A, 80–89 points for B, 70–79 points for C, and an F for points 69 and below [13,14].

## 3. Results

Course duration: In the current study, the format of didactic instruction in the pathology courses included thirty-two hours of lectures and online activities and sixty-four hours of individual student–instructor hours as needed. Each year, students were assessed by four MCQ tests over the course of 16 weeks. Table 1 gives examples of MCQs covering two distinct concepts of the oral pathology curriculum.

The reliability of exams: The reliability of the examination was measured using KR-20; a high KR-20 indicates that if the same exam takers took the same assessment, there is a higher chance that the results would be the same. A low KR-20 means that the results would be more likely to be different [12]. A KR-20 value of <0.3 is considered poor, and a value of ≥0.7 is considered acceptable [15,16]. The mean and standard deviation of the KR-20 value for each of the four exams administered over five consecutive years is given in Table 2.

The item analysis of exams: The mean DI of the examinations ranged from 63% in 2018 to 81% in 2015, that of the mean Disc-I ranged between 0.25 in 2019 to 0.43 in 2016, and that of the mean point biserial ranged between 0.25 in 2015 to 0.45 in 2016 (Table 3). The mean DI for all of the exams for the course in each year was 72 +/− 4.72%, a value that is widely considered acceptable. The overall mean Discriminatory Index was 0.33 +/− 0.05, and the overall mean point biserial was 0.34 +/− 0.05 (Table 2). The average DI was significantly higher in the year 2015 (77.8 +/− 2.9%) than that for the years 2017 (70.3 +/− 3.3%), 2018 (71.3 +/− 5.4%), and 2019 (70 +/− 3.54%). The Discriminatory Index and point biserial were significantly lower in 2017 compared to the values in 2015.

**Figure 1:**
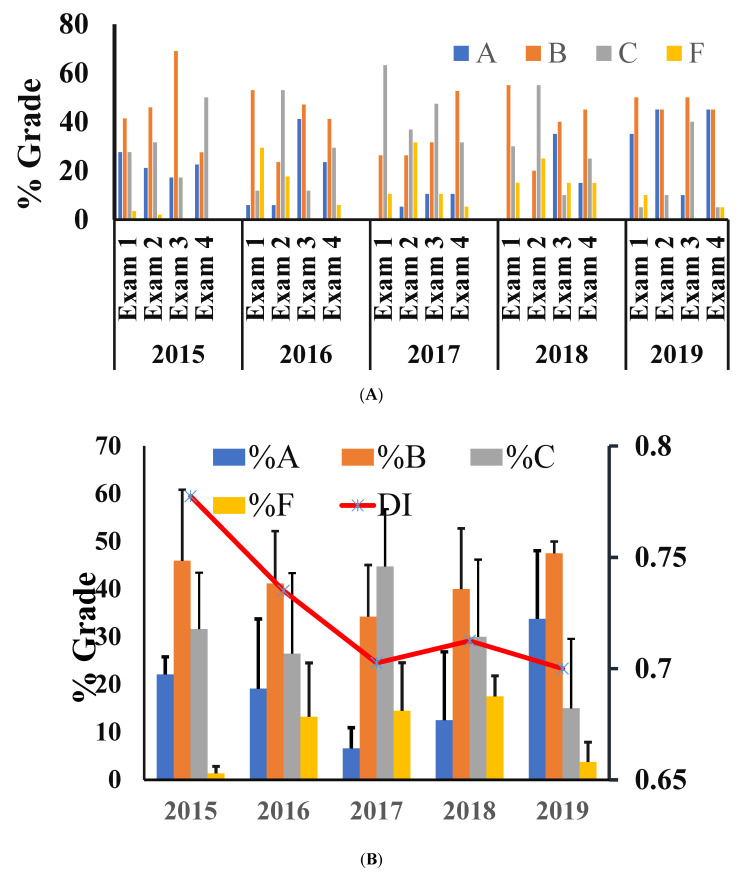
(**A**) Letter grade in each exam for each year. The students were provided a letter grade based on the percentage of their raw score against a fixed scale of absolute grading: 90–100% for A, 80–89% for B, 70–79% for C, and an F for points 69% and below. (**B**) The average of the four exams for each year was used as the final grade for the oral pathology course. The line graph is the average Discriminatory Index for all four exams of the indicated year.

Course grade across five years: The exam takers were provided an absolute grade based on predetermined cutoff levels. Data showed that the letter grade A was achieved from 20% to 35% of exam takers in each of the four exams in 2015 and 2019. The letter grade B was the most common, obtained by >50% of the exam takers in all years except 2017, in which the common grade of exam takers was C (Figure 1A). The final grade for the course based on the average of four exams per year suggested that the most common grade for the course was B for all years except for 2017, with most exam takers obtaining a C. The percentage of exam takers with a grade F was higher in the years 2016–2018.

**Figure 2:**
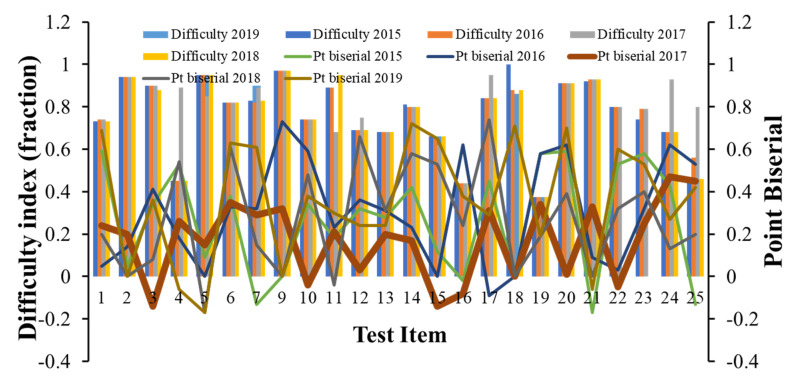
The relationship between the DI and point biserial of test items. Twenty-five test items included in the exams in each year with a similar DI were selected. Line graphs are the point biserial value of the indicated test item. The point biserial value (the broad brown line) indicates the values of the test time in 2017.

Relationship between grade distribution and DI and Disc-I: As noted above, although the material taught, the instructor, and the multiple-choice question developer were the same across the years, the distribution of grading was different. The DI of 78% suggested that the 2015 cohort of exam takers found that the test items were relatively easy, and was reflected in the higher percentage of individuals achieving the letter grade A in 2015 (Figure 1B). The lower Disc-I and point biserial in the 2017 cohort suggested that the items were identified as relatively hard, and thereby was reflected the lower percentage of exam takers who achieved scores consistent with the letter grade A (Figure 2) [17].

Next, the contribution of individual item characteristics, DI, Disc I, or point biserial, to the differences in the grade distribution was evaluated. Analysis of performance of twenty-five test items with approximately the same DI ( +/− 0.05) showed that the test items exhibited varied Disc-I and point biserial indices, potentially reflecting the differences in the ability of the exam taker cohorts (Figure 2).

## 4. Discussion

One of the most challenging areas in dental and dental hygiene practice is the diagnostic process. The dental hygienist plays a key role in the preliminary evaluation and data collection of oral pathologic conditions for final diagnosis and management by the dentist [18]. Instruction in pathology content areas helps prepare the students of dental hygiene for this role. It has been stated that the knowledge gained from pathology instruction enables students to understand and participate comprehensively in the delivery of healthcare [3].

In addition to the content, the assessment of learning is an important element of an instructional design process. It provides feedback on learning and teaching processes and enables the review and improvement of the whole process [5,19]. Although some basic test statistics, including the mean, median, and mode, have been carried out routinely, there is a paucity of item analysis in specific subjects of the pathology course domain in dental hygiene education. This study examined the assessment of the oral pathology instructions in the dental hygiene program from two different perspectives: evaluating student learning using item analysis of MCQs and evaluating the test content in assessing student comprehension across multiple years. Furthermore, we discuss whether the data could be of value in a retrospective analysis of admission criteria to the dental hygiene program.

This study included only single best response MCQs designed specifically to assess broad domains of knowledge effectively and reliably [15,20,21]. In the present study, the mean DI value was 72.6 +/− 4.7%, which is widely considered an acceptable level of difficulty. Similarly, the mean Disc-I and point biserial values, 0.33 +/− 0.05 and 0.34 +/− 0.05, respectively, were also within an acceptable range. The Disc-I and point biserial coefficient values correlate with a dichotomous variable (a right or wrong response for a single item) and a continuous variable (the test score) [15,22,23]. The data showed that these values exhibited significant variations for items with similar levels of DI in different cohorts of exam takers. Although variations in discriminatory indices are often considered indicators of ambiguous wording, the wide scatter could also reflect some extent of guessing practices [8,24,25]. Pertinently, Dascalu et al. observed that, in a cohort of students in dental medicine, while the grades of MCQ tests followed a normal distribution, the average was significantly lower than that in traditional oral examinations [6]. A limitation of this study is that the analysis was restricted to one subject domain in the senior year of the dental hygiene curriculum, and may not be representative of the overall ability of the exam takers to succeed in other didactic or clinical courses.

Alternatively, variations in the Discriminatory Index and point biserial across the years could reflect the learning attributes of the exam taker cohort. It is observed that the analysis across five years showed that the Discriminatory Index for the same test item was consistently lower in the year 2017, and the average test grade for this cohort was one grade lower (C) than that of the other years. In this context, it is interesting to note that the KR-20 value for exams three and four in the year 2017 were 0.57 and 0.56, respectively. Since the calculation of KR-20 is based on the standard deviation and the proportion of students responding correctly and incorrectly, it appears that the lower Discriminatory Index for the year 2017 could at least be partially cohort specific. Typically, admissions to the dental hygiene program require at least one year of college and the completion of prerequisite courses, including anatomy, biology, chemistry, microbiology, and math. Interestingly, in a recent analysis of predictive factors for student success in dental school, Sabato et al. showed that the elements of undergraduate education could help identify students who are at risk for poor performance and require timely intervention [26].

## 5. Conclusions

As with other health professional training, the effective measurement of knowledge is an important component of both allied dental education and practice [20,27,28]. Well-designed, single-choice MCQs are excellent tools to align the evidence resulting from the tests with student achievement or cognition as reflected in the grades [4,15]. Our observations of a low-achieving cohort amongst the cohorts of five consecutive years evaluated for the same knowledge domain using similar test tools suggest that the comparative analyses of tests could offer some feedback not only on learning abilities, but also on the selection processes for admission to the dental hygiene program. It will be interesting to analyze similarly concurrent courses offered and tested for the same cohort of exam takers.

## Figures and Tables

**Table 1:** Examples of MCQs and respective DI and Disc-I.

A 50-year-old ex-smoker is referred to the dentist by a cardiologist. Past history includes severe recurrent oral ulcerations affecting lateral borders of the tongue, labial mucosa, and soft palate. Ulcers are one or two at a time and persist for about eight weeks. Medical history showed use of a potassium channel activator (nicorandil) for unstable angina and aspirin (75 mg/day) since his myocardial infarction nine months ago. He has no eye, skin, or genital ulcerations. The most probable cause of the major RAS ulcers is		Aspirin burn Bechet’s disease Nicorandil use (potassium channel blocker) Smoking cessation
**Academic Year**	**DI**	**Disc-I**
2015	0.65	0.4
2016	0.65	0.6
2017	0.63	0.6
2018	0.1	0.2
2019	0.35	0.0
A middle-aged man presented with a slowly growing swelling on the left side of the mandible. The X-ray showed driven snow appearance of mixed radio-opacity and radiolucency. The most likely diagnosis is		Odontogenic keratocyst Ameloblastoma Pindborg’s tumor Compound odontoma
**Academic Year**	**DI**	**Disc-I**
2015	0.72	0.38
2016	0.76	0.4
2017	0.79	0.2
2018	0.75	0.4

**Table 2:** The reliability score of each exam as determined by the Kuder–Richardson formula 20 coefficient (KR-20).

	KR-20 Values						
	2015	2016	2017	2018	2019	Average	SD
Exam 1	0.73	0.68	0.7	0.71	0.76	0.716	0.03
Exam 2	0.72	0.52	0.81	0.68	0.76	0.698	0.10
Exam 3	0.77	0.77	0.57	0.77	0.68	0.712	0.08
Exam 4	0.7	0.68	0.56	0.83	0.67	0.688	0.09
Average	0.73	0.6625	0.66	0.7475	0.7175		
SD	0.03	0.09	0.10	0.06	0.04		

**Table 3:** Item analysis including the Difficulty Index, Discriminatory Index, and point biserial score of each exam.

**A**	**Difficulty Index**							
		**2015**	**2016**	**2017**	**2018**	**2019**	**Average**	**SD**
	Exam 1	0.78	0.71	0.67	0.7	0.73	0.72	0.04
	Exam 2	0.73	0.7	0.67	0.63	0.67	0.68	0.03
	Exam 3	0.79	0.77	0.74	0.77	0.66	0.75	0.05
	Exam 4	0.81	0.76	0.73	0.75	0.74	0.76	0.03
	Average	0.78	0.74	0.70	0.71	0.7	0.73	0.03
	SD	0.03	0.03	0.03	0.05	0.04		
**B**	**Discriminatory Index**							
		**2015**	**2016**	**2017**	**2018**	**2019**	**Average**	**SD**
	Exam 1	0.35	0.43	0.29	0.29	0.25	0.32	0.06
	Exam 2	0.3	0.33	0.37	0.41	0.37	0.36	0.04
	Exam 3	0.35	0.34	0.26	0.35	0.26	0.31	0.04
	Exam 4	0.28	0.4	0.3	0.34	0.31	0.33	0.04
	Average	0.32	0.375	0.305	0.35	0.3		
	SD	0.03	0.04	0.04	0.04	0.05		
**C**	**Point Biserial**						**Average**	**SD**
	Exam 1	0.34	0.4	0.25	0.31	0.28	0.32	0.05
	Exam 2	0.35	0.32	0.35	0.34	0.33	0.34	0.01
	Exam 3	0.32	0.45	0.27	0.37	0.32	0.34	0.06
	Exam 4	0.33	0.43	0.32	0.36	0.33	0.35	0.04
	Average	0.34	0.4	0.3	0.35	0.32		
	SD	0.01	0.04	0.04	0.02	0.02		
